# Evaluation of Thermomechanical Properties of Polymer Blends Intended for Additive Manufacturing Processing: Comparative Study of Poly(Lactic Acid) and Poly(Lactic Acid)/Polyhydroxyalkanoate Blends

**DOI:** 10.3390/polym17182454

**Published:** 2025-09-10

**Authors:** Jacek Andrzejewski, Katarzyna Skórczewska, Mateusz Barczewski

**Affiliations:** 1 Institute of Materials Technology, Faculty of Mechanical Engineering, Poznan University of Technology, Piotrowo 3 Street, 61-138 Poznan, Poland; mateusz.barczewski@put.poznan.pl; 2 Faculty of Chemical Technology and Engineering, Bydgoszcz University of Science and Technology, Seminaryjna 3, 85-326 Bydgoszcz, Poland; katarzyna.skorczewska@pbs.edu.pl

**Keywords:** polymer blends: poly(lactic acid), polyhydroxyalkanoates, additive manufacturing, thermal resistance, mechanical performance

## Abstract

The following article presents the results of research on the assessment of the effect of FDM printing process conditions on the properties of the obtained products. During the research, unmodified PLA and a PLA/PHA mixture were subjected to comparative analysis. Both materials exhibit excellent processability under standard FDM process conditions; however, the PLA/PHA blend is gaining attention due to its potential as a more thermally resistant and less brittle alternative to unmodified PLA. The printing procedure conducted at high bed platform temperature confirmed that for standard PLA varieties and the PLA/PHA blend, it is possible to obtain improved thermomechanical properties only by modifying the machine parameters of the printing process. The increase in the Vicat softening temperature value was about 80 °C, reaching above 130 °C. Interestingly, for materials based on pure PLA, most mechanical properties exhibit noticeable improvement, with the improvement in impact strength being particularly beneficial. For most materials, the measurements revealed significant anisotropy of properties within the tested samples, which was particularly due to the use of different bed platform temperatures. The apparent effect of this was the change in the thermal conditions of the PLA phase crystallization process, with crystallinity levels ranging from 17 to 33% for selected samples. The obtained results confirm that PLA/PHA blends are an interesting alternative for the PLA-based material; however, further research is needed to improve the application potential further.

## 1. Introduction

The use of PLA-based materials in filament-based 3D printing processing is one of the most popular solutions for unprofessional users when starting with rapid prototyping or small-scale manufacturing. Unfortunately, due to its properties, pure PLA is not a good example of a technical polymer, the main limitations are high brittleness and low resistance to elevated temperatures. Of course, the above factors limit the use of PLA not only in 3D printing techniques, but also as a material for packaging production or as a material intended for injection molding of components in the automotive or household appliances industry. For many applications involving mass use of extrusion or injection technology, PLA is a niche material that is very rarely filled. However, due to the growing interest of consumers in the ecological balance of polymer products and their management after the product lifetime, PLA, as a bio-based and biodegradable material, is gaining popularity in many manufacturing areas.

In the research conducted so far, various concepts have been proposed for enhancing the properties of PLA-based products, with the most straightforward approach being the production of a polymer blend [1,2,3]. In this case, the simplest method of modification is the addition of technical materials with the desired properties for a given application. In the research conducted so far, numerous examples of materials based on PLA with the addition of high-crystalline polymers, such as polyamides (PA) or polyoxymethylene (POM), have been reported [4,5,6,7], where the primary reason for the blending procedure was to enhance the thermal resistance of the resulting compound [8,9]. In this case, the best results are obtained for PA11-based materials, due to the similar melting point to PLA, which reduces problems with material degradation. Another group of polymers tested for their potential use as PLA-blend ingredients was thermoplastic polyesters, such as polyethylene terephthalate PET or polybutylene terephthalate PBT [10,11,12]. Despite PLA belonging to the same group of materials, studies on PLA/PET systems indicate minimal potential for this type of material. In the case of mixtures of the discussed type, it is still possible to transfer numerous samples, combinations of PLA-based systems with polymers such as ABS, PMMA, or PC [13,14,15,16,17,18]; however, these studies clearly indicate that only for a few mixtures is it possible to obtain favorable material properties. unfortunately, this is usually possible for systems containing a relatively low PLA content [19,20,21].

In the context of using PLA-based blend systems, a much more effective strategy is to use the addition of elastomers or soft polymer varieties. In this case, an additional advantage of this solution is the possibility of utilizing a broad range of bio-based and biodegradable materials. Among the research works conducted so far in this category of materials, the most numerous works concerned the use of polybutylene adipate terephthalate PBAT [22,23,24] or polycaprolactone PCL [25,26,27] additives. It is worth emphasizing that the materials in question have a considerable potential for industrial applications, confirmed by the commercialization of such commercially available materials as Ecovio^®^ or Materi-bi [28,29,30,31]. It is worth noting that the primary effect of using PLA blends with soft polymers is an increase in elongation and impact strength, while issues related to low thermal resistance typically remain at a similar level. Therefore, in this respect, for the discussed research, it was decided to adopt a combined approach that incorporates both material and process modifications.

The previous work on PLA modification to improve the mechanical properties of this polymer indicates the key importance of crystallinity. Most studies suggest that with an appropriately high crystallinity, it is possible to achieve satisfactory thermal resistance (HDT/Vicat test results) at temperatures as high as 150 °C [32,33]. However, this is only possible when using an appropriate nucleating system, with a high filler content of mineral/fibrous fillers, and possible a slow cooling procedure and ensuring sufficiently slow crystallization at high temperatures, which usually is impossible or inefficient for most industrial processes [34,35,36,37]. An alternative approach is to use the annealing procedure. This time, the procedure can be treated as an additional process, performed as post-processing; however, the danger associated with it concerns the potential change in the shape of the products during the initial stage of the heating, when the amorphous PLA structure softens at low temperature (above 60 °C). While the positive stiffness increases, due to the rise in the content of crystalline structures, usually takes place above 100 °C [38,39].

For the concept of FDM printing at elevated temperature presented in the paper, the PLA/PHA system was used, a commercially available variant of which was used in the sample preparation process. This material has been treated so far as a potential replacement for standard PLA. The results of previous work do not indicate any particular differences in functional properties for this type of material, which results in moderate market interest in this type of material. The results of previous work only indicate an increased potential for degradation in composting conditions [28,40], which is a relatively moderate factor for end users. However, upon detailed analysis of the results, specific differences in the properties of the PLA/PHA mixture become apparent, particularly a potentially faster crystallization process, which may indicate an advantage for these materials in competition with PLA.

For the discussed research work, the primary goal was to prepare samples at an elevated temperature on the bed platform, allowing for thermal conditions that facilitate the growth of the PLA crystalline phase. The maximum process temperature of 115 °C was limited by the 3D printer device’s capacity, which is the typical limit for desktop-type machines. To reveal the influence of the printing process conditions, the basic mechanical properties were evaluated during the static tensile and flexural measurements, and for the impact resistance, we used the notched Izod tests. The thermomechanical properties were compared using standardized heat deflection temperature (HDT) and Vicat softening (VST) methods, while the viscoelastic properties, like storage modulus and tan δ damping factor, were evaluated using dynamic mechanical thermal analysis (DMTA). The phase transitions and crystallinity evaluation were performed with the differential scanning calorimetry method (DSC), while the structure analysis was supplemented with wide-angle X-ray diffraction measurements (XRD). The internal structure differences were analyzed using scanning electron microscopy (SEM) observations.

## 2. Experimental Methodology

### 2.1. Materials

The prepared materials were supplied in the form of ready-to-use filaments by technovAde (Poznan, Poland). The first type of filament was pure, unmodified poly(lactic acid) under the trade name PLA Natural (1.75 mm), manufactured by Devil Design (Katowice, Poland). The second type of samples was prepared from the commercially available blend declared as PLA/PHA composition (colorFabb company, Belfeld, The Netherlands). Previous studies have characterized this type of composition as a blend composition, containing 88% of the PLA phase with a 5% *D*-lactic content. More complex composition refers to the PHA compound, where most of the studies confirmed that the blend is mainly composed of 3-hydroxybutyrate with some addition of 3-hydroxyvalerate [41,42,43].

### 2.2. Sample Preparation

**Figure 1:**
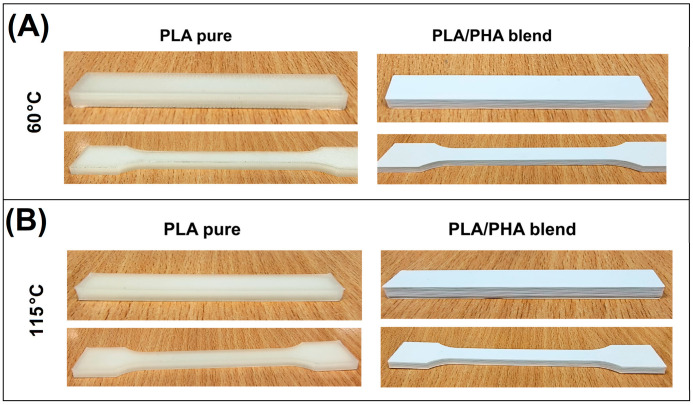
The appearance of the PLA and PLA/PHA testing specimens prepared at different bed platform temperatures: (**A**) 60 °C and (**B**) 115 °C.

The sample preparation was limited to 3D printing processing, since the supplied materials were already in filament form (diameter of 1.75 mm). The equipment we used was a desktop-type printer, Prusa MK3S (from PrusaResearch, Prague, Czech Republic). The testing coupons were prepared in accordance with ISO 527-1:2019 standard, [44] requirements using generated STL files. For other tests, the rectangular bars were prepared (80 mm × 10 mm × 4 mm). For all of the prepared samples, the extruder nozzle temperature was 210 °C, while the bed platform temperature was set to 60 °C, 90 °C, and 115 °C. The other parameters were fixed again; the printing speed for parameter layers was set to 40 mm/s, while the infill structure was printed with 80 mm/s. Before each print, the surface of the bed platform was covered with a thin layer of adhesive glue Dimafix (i3D, Almeria, Spain). In the case of any of the produced series of samples, no significant problems resulting from the occurrence of shrinkage and buckling phenomena were observed. The samples, apart from some differences in discoloration, did not differ significantly from one another (see Figure 1).

### 2.3. Characteristics

The mechanical properties of the prepared samples were evaluated using static and dynamic methods. For static load mode, we use tensile/flexural testing methodology. For the tensile tests, the measurements were performed in accordance with the ISO 527 standard. The 1A type specimens (gauge distance 50 mm) were stretched with the cross-head speed of 10 mm/min. In order to correctly determine the module value, the initial stage of measurement was carried out at a speed of 1 mm/min. Displacement values were read directly from the machine; no extensometer was used. For the flexural tests, the measurements were carried out in accordance with the guidelines of the ISO 178:2019 standard [45]. The rectangular bars (80 mm × 10 mm × 4 mm) were used as the testing specimens, where the span distance was 64 mm. The cross-head speed was set to 100 mm/min, while, similar to the tensile tests, the initial deformation rate was reduced to 1 mm/min. All tests were performed using Zwick/Roell Z010 universal testing machine (Zwick/Roell GmbH, Ulm, Germany), which was equipped with a 10 kN load cell. The final results were calculated from the average recorded from at least 5 samples.

The impact resistance measurements were conducted using the Izod method. The printed samples were notched using a dedicated cutting knife; the notch depth was 2 mm, and the notch angle was 45°. The testing procedure was conducted in accordance with the ISO 180:2023 standard, [46]. The tests were performed using a Zwick/Roell HIT15 machine (Zwick/Roell GmbH, Ulm, Germany) with a pendulum energy of 5 J.

The thermal properties and phase region changes were investigated using the differential scanning calorimetry method (DSC). The small 5 mg specimens were placed inside the pierced aluminum crucibles. The heating/cooling rate was set to 10 °C/min, while the scanning range was set to 22–225 °C. The standard heating/cooling/heating procedure was applied. The oven chamber was purged with a protective nitrogen atmosphere, flow rate 20 mL/min. The tests were conducted using the Phoenix DSC 204 F1 apparatus (Netzsch GmbH, Selb, Germany).

The thermal stability of the used filaments was evaluated using the thermogravimetric (TGA) method. The tests were conducted using the standard apparatus model TG 209 F1 Libra (Netzsch GmbH, Selb, Germany). Tests were performed using standardized methodology, where a small sample of 10 mg was placed inside the ceramic crucible and apparatus chamber. Tests were conducted from the room temperature to 700 °C, where the heating rate was set to 10 °C/min. The procedure was performed under a protective nitrogen atmosphere, with a gas flow of 20 mL/min.

The thermomechanical evaluation of material properties was performed using dynamic mechanical thermal analysis (DMTA), where basic viscoelastic properties were recorded during the temperature scan. For all of the tested materials, the test temperature ranged from 30 °C to 150 °C, while the heating rate was 2 °C/min. The deformation of 0.01% was applied with a frequency of 1 Hz. The tests were conducted using the MCR 301 apparatus (Anton Paar GmbH, Graz, Austria), which was equipped with a torsion mode clamp system. The thermomechanical analysis was supplemented with industrial-type measurements, heat deflection temperature (HDT), and Vicat softening temperature (VST). Both tests were conducted using an HDT/Vicat HV300C machine (Testlab, Warsaw, Poland). The machine was equipped with an oil bath, while the heating rate for the conducted test was 2 °C/min. For the HDT test, the applied load was 1.8 MPa, while for the Vicat testing, it was 10 N.

The internal structure of the manufactured materials was investigated using the scanning electron microscopy method (SEM). For this purpose, the fractured surface of the impact test specimen was covered with a thin layer of gold using a plasma sputtering machine. The surface scanning was performed using an EVO 40 SEM microscope (Carl Zeiss, Jena, Germany).

The structural studies will be supplemented by wide-angle X-ray diffraction measurements using a URD 6 diffractometer from Rich Seifert & Co. GmbH (Freiberg, Germany). During the measurements, monochromatic X-ray diffraction with a wavelength of λ = 1.5406 Å (CuKα) was used in the 2θ angle range from 10° to 75° with a step size of 0.05.

## 3. Results and Discussion

### 3.1. Thermal Stability of Filament Materials—Thermogravimetric Analysis (TGA)

**Figure 2:**
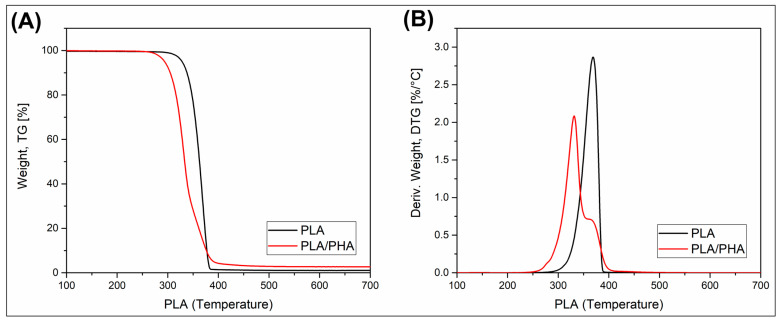
The results of the thermogravimetric analysis (TGA) for unprocessed filament samples. The results are collected in the form of (**A**) weight loss plots (TG) and (**B**) derivative weight plots (DTG).

Since the research concept was mainly focused on the evaluation of the mechanical and thermomechanical performance of the sample, the TGA analysis was performed as a supplemental study. The primary objective of this study was to identify the fundamental differences in thermal stability between pure PLA and PLA/PHA materials, which is why only filament samples were used for research. The TG and DTG thermograms are presented in Figure 2.

It is clear that the thermal resistance of pure PLA is higher than that of PLA/PHA materials. The initial 5% weight loss for PLA was recorded at around 330 °C, whereas for the blended PLA/PHA specimen, it was recorded at 290 °C. These results are firmly in line with the previous reports [43]. It is also worth noting that the final stage of decomposition, where the weight loss reached a minimum, was close to 390 °C for both types of material. Interestingly, the previous reports confirm that the PHA phase decomposition takes place close to 300 °C, with a significant amount of char residue (≈10%). In our study, the char residue for PLA was 1%, while for the PLA/PHA sample, around 2.5%, which cannot be considered significant, suggesting the presence of an inorganic compound or carbonization of the sample compounds. The two-stage decomposition of the PLA/PHA specimens is directly associated with the difference in decomposition temperature for the PLA and PHA phases. Interestingly, the kinetics of the initial stage of decomposition were higher, which suggests that the PHA decomposition products accelerate the decomposition of the PLA phase, which is a fairly common phenomenon for polymer blend systems.

### 3.2. Mechanical Properties Evaluation—Static Tensile/Flexural Tests, Izod Impact Resistance Measurements

**Figure 3:**
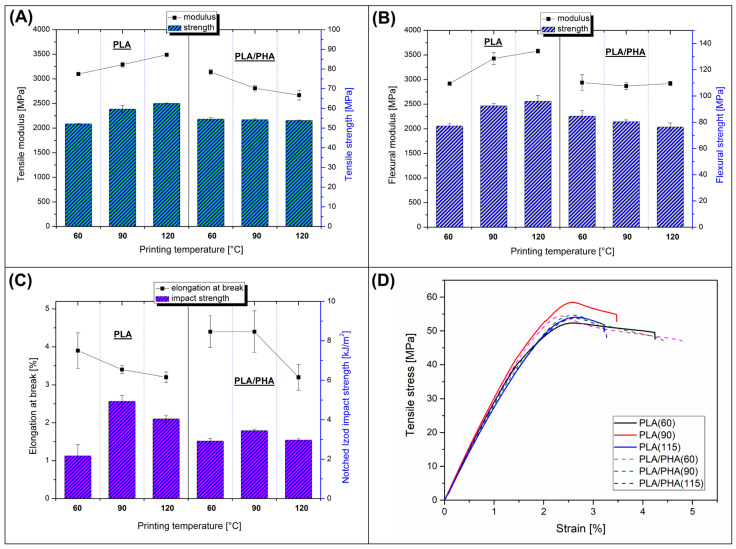
The results collected from the static tensile/flexural measurements and Izod impact resistance tests: (**A**) tensile modulus/strength comparison; (**B**) flexural modulus/strength results; (**C**) elongation at break/impact strength; (**D**) the stress–strain curves for all of the PLA and PLA/PHA samples.

The mechanical properties of the prepared samples are collected in Figure 3, where the static tensile/flexural test results are combined with the notched Izod impact strength results. The most important mechanical characteristics of pure PLA and PLA/PHA materials were measured for samples printed at different bed platform temperatures (60 °C, 90 °C, and 115 °C). The results are supplemented with stress–strain plots for all samples (Figure 3D).

The results of the static measurements revealed that for pure PLA samples, the increasing bed temperature has a slight influence on the resulting strength and modulus values in both tensile and flexural tests. For example, the recorded values for flexural modulus were approximately 2900 MPa for the sample with a 60 °C bed temperature, while for PLA printed at 115 °C, the stiffness was approximately 3600 MPa. A similar visible difference was observed for flexural strength values, where for the same group of specimens, the recorded values were 75 MPa and 95 MPa, respectively. For the tensile test results, the trends for PLA materials were very similar. Interestingly, for the PLA/PHA blend, the positive bed temperature influence was not confirmed. In fact, most of the strength/modulus results were constant regardless of temperature. For example, the tensile strength values were close to 55 MPa, while for flexural strength, the values ranged from 85 MPa to 75 MPa. That means the increasing bed platform temperature is not leading to strength/stiffness improvement in PLA/PHA blend; in some cases, like tensile strength changes, the increased bed temperature was leading to visible deterioration of mechanical properties.

Besides the strength/modulus results, the tensile testing measurements allow for an analysis of changes in elongation at break. In general, for both pure PLA and PLA/PHA, the maximum elongation values were relatively low, which is not surprising for unmodified PLA (≈4%); however, the results for the PLA/PHA (≈4.5%) might be somewhat disappointing. In the case of both types of materials, the increasing bed temperature is leading to a decrease in visible elongation, where for samples printed at 115 °C, the resulting elongation at break was limited to 3%, for both pure PLA and PLA/PHA samples.

In most cases, where polymer stiffness values are improved due to some structural reinforcement, the result is limited elongation and reduced impact resistance. However, that assumption is not valid for many semi-crystalline polymers, where the increased modulus results from the higher level of crystallinity. In contrast to reduced elongation at break values, the impact resistance can be visibly improved, which is usually associated with the ligament thickness-related toughening mechanism [47,48]. In general, the effectiveness of this phenomenon depends on the morphology of the crystalline phase, where it is beneficial when the increase in the level of crystallinity of the material leads to the physical connection of the PLA lamellar structures. For materials with too low a level of crystallinity, the distances between the spherulite structures are too large to achieve this effect. However, for polymer mixtures, the increase in the PLA crystalline phase around the inclusion of the dispersed phase often leads to a decrease in the required ligament thickness, increasing the impact strength of the material. The effectiveness of this type of mechanism has been confirmed for many types of thermoplastic polymers, such as PP and PE, while for low-crystalline polyesters, including PET and PLA, additional heating is often required. For the reference PLA(60) samples, where the material was printed at 60 °C, the impact strength value was close to 1.2 kJ/m^2^. For the PLA(90) and PLA(115) materials, the recorded strength values were 2.6 kJ/m^2^ and 2.1 kJ/m^2^, respectively. That trend confirms the presence of a crystallinity-related toughening mechanism. As confirmed by other research studies, even for unmodified PLA, the impact strength can be significantly improved by successful crystalline structure nucleation or annealing [49,50,51,52,53,54]; however, for higher efficiency, the procedures should be combined with the addition of impact modifiers, like elastomeric or soft polymer phase [22,23,47,48,55,56,57].

Interestingly, for PLA/PHA materials, increasing the bed temperature did not lead to visible changes in toughness. The results were in the range of 1.5 kJ/m^2^ and 1.8 kJ/m^2^. The presence of the secondary phase should lead to the formation of a double-phase structure; however, for the examined blends, we noticed that the clear two-phase structure was not present for the investigated type of blend. The possible reason for that was the improved miscibility of the PLA/PHA system. This conclusion was confirmed by microscopic observations, where, even with high magnification, a clear distinction between the phase inclusions was not possible. This fact does not exclude the presence of a highly fragmented form of the phase system; however, even such a situation suggests the occurrence of partial miscibility in the mixture. Despite some doubts regarding the probable reasons for the lack of significant changes in mechanical properties for PLA/PHA materials, it is worth noting the absence of clear downward trends, where an increase in the temperature of the 3D printing process would lead to a deterioration in the functional properties of the finished products.

Summarizing the obtained results, it is clear that for unmodified PLA, the increased bed temperature led to favorable changes in mechanical performance, which phenomenon is mainly associated with an increased level of crystallinity. Another possible factor that could improve the properties was the interlayer fusion strength. However, considering that for both tensile and flexural test specimens, the delamination phenomenon was not present in any of the samples, interlayer strength was a relatively negligible factor in standardized measurements. Unlike pure PLA, the mechanical performance of PLA/PHA blend was only slightly influenced by the bed platform temperature.

### 3.3. Thermomechanical Properties—Dynamic Mechanical Analysis (DMA), Heat Deflection/Vicat Softening Temperature Evaluation (HDT/VST)

**Figure 4:**
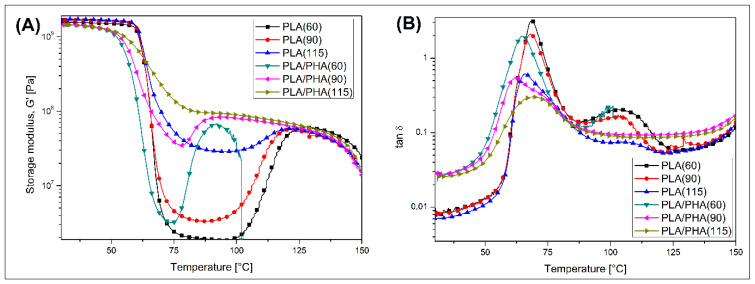
The results of the DMTA for PLA and PLA/PHA materials: (**A**) storage modulus; (**B**) tan δ plots. Plots represent the specimens printed at different bed platform temperatures (60 °C, 90 °C, and 115 °C).

**Figure 5:**
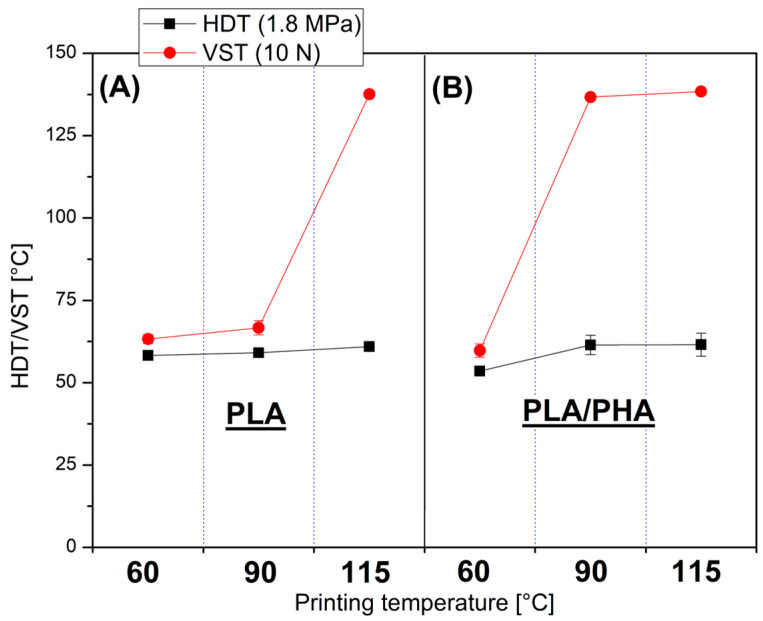
The thermomechanical test results, heat deflection temperature HDT, and Vicat softening temperature (VST) for (**A**) pure PLA samples, and (**B**) PLA/PHA blend.

The thermomechanical properties of the prepared samples were investigated using three techniques, where dynamic mechanical analysis (DMTA) was used as the main analytical method (see Figure 4), while heat deflection tests (HDT) and Vicat softening temperature method (VST) were used as supplementary techniques (see Figure 5). The DMTA was performed from the room temperature range up to 150 °C; however, due to intensive softening issues, the test for the PLA/PHA(60) sample was terminated at around 100 °C.

The storage modulus recorded for pure PLA specimens shows clearly that the glass transition phenomenon leads to a rapid change in stiffness. The sharpest drop was observed for the reference PLA(60) specimen, which confirmed the presence of an almost fully amorphous structure of the macromolecules. The presence of a highly amorphous structure in PLA(60) samples is confirmed by analyzing the tan δ plots, where the glass transition peak position for the reference material is the highest among the entire group of tested samples. As can be predicted, the changing of the bed platform temperature also influences the conditions of the crystallization process. Two possible mechanisms are present here, where the first one is associated with the slowing down of the cooling rate of the deposited polymer layers, while the second one is related to the phenomenon of cold crystallization occurring above the glass transition temperature. It is worth pointing out that for the FDM/MEX printing process, subsequent layers of the model are cooled by air flow, where its intensity does not change with the change in the table temperature. It should therefore be assumed that the cooling intensity (rate) of the individual layers of the model is relatively similar, despite the change in the table temperature; the exception here is the first layer, the deposition of which takes place with the fan turned off [58,59]. It can therefore be assumed that the phenomenon of cold crystallization has a much greater influence on the increase in the level of the crystalline phase, and thus also on the change in viscoelastic properties. The storage modulus plots for pure PLA indicate the appearance of direct changes in the E’ modulus values at about 100 °C, which would suggest that for printed PLA (90) materials, i.e., slightly below the temperature, this transformation occurred in the reference sample. However, it should be remembered that in the case of DMTA measurements, the registration of phase changes depends on the sample size and, therefore, its thermal inertia. At a thickness of 4 mm, changes in the mobility of the macromolecular structure require much more time than during DSC studies, where the sample mass is about 5 mg. It can therefore be assumed that already at a temperature of 90 °C the influence of the bed table temperature is significant enough to induce cold crystallization effects, while the change in viscoelastic properties is only small here and limited to a visible increase in the storage modulus value in the glass transition range and a slight reduction in the tan δ peak height.

Further increasing the bed temperature to 115 °C results in an even more significant improvement in thermomechanical stability. The effect of structural changes is visible in particular through the flattening of the glass transition region, where the E’ modulus curve almost smoothly changes from the values recorded at room temperature to a relatively stable range above 120 °C. The observed changes clearly indicate an increase in the content of the PLA crystalline phase, which is additionally confirmed by a significant decrease in the tan δ peak value.

Considering that for pure PLA, the significant changes in viscoelastic properties are observed for the PLA(115) sample, the plot analysis for PLA/PHA-based materials revealed that starting from 90 °C bed temperature, the thermal resistance of the prepared materials became visibly higher. However, even for the reference material PLA/PHA (60), the recorded storage modulus values suggest that the beginning of the cold crystallization temperature area is shifted to a significantly lower temperature, close to 75–80 °C, rather than 90–100 °C, as is the case for unmodified PLA. That behavior clearly confirmed that for the used PLA/PHA blend, the crystalline structure of the PLA phase is nucleated by the presence of PHA phase inclusions. Interestingly, after increasing the bed temperature to 80 °C, the discussed stiffness increase was visibly limited, which is clearly related to the overall rise in the E’ modulus value, especially in the glass transition range. For the PLA/PHA (115) sample, the cold crystallization phenomenon was not observed; the decrease in storage modulus value is minimal. Those observations are strongly in line with the other test results, where, for most of the results, the presence of PHA inclusions significantly influences the formation of the PLA crystalline structure.

The DMTA is supplemented with heat deflection/Vicat softening temperature measurements (HDT/VST), where both types of thermomechanical evaluation were performed for each group of specimens. Similarly to DMTA, where increasing bed platform temperature was leading to visible changes in storage modulus plots, the thermomechanical tests are also reflecting the same trends; however, due to the measurement simplicity HDT and VST measurement results only indicate point changes in the form of significant increase in the deflection or softening temperature, while DMTA graphs allow for tracing the scale of changes in a more complex way. Generally, the results of HDT tests, in the form of recorded deflection temperatures, are lower than those of VST tests, where a blunt needle tip actually pierces the sample. Additionally, considering the issue of structural anisotropy, it is worth mentioning that for polymer blends, unlike fiber-reinforced composites, changes in structural orientation have a negligible effect on thermomechanical properties.

Interestingly, for HDT measurements, the correlation of the bed platform temperature and thermal resistance is almost negligible. For PLA(60) and PLA (115) sample the HDT(1.8 MPa) was 58 °C and 61 °C, respectively. Similarly, the difference between PLA/PHA(60) and PLA/PHA(115) material was small, respectively, 54 °C and 62 °C. For both types of samples, the small upward trend is visible; however, it is clear that the change in thermal conditions does not increase heat deflection temperature limits. More promising data are collected when analyzing the Vicat softening temperature results. The significant improvement for pure PLA materials was possible only for the PLA(115) sample, where the VST value reached 138 °C. Similar improvements were observed for PLA/PHA materials at bed temperatures of 90 °C and 115 °C, where the Vicat temperature again reached 137 °C and 138 °C. The obtained results suggest a significant difference in thermal resistance performance; however, it should be noted that for the HDT test, the applied load was relatively high, which suggests that for a lower stress of 0.455, the thermal resistance might be slightly improved. However, the verification carried out still indicates a better potential for high-temperature applications for the PLA/PHA blend, which was the main goal of the conducted measurements.

### 3.4. Internal Structure Analysis—Scanning Electron Microscopy Observations (SEM)

**Figure 6:**
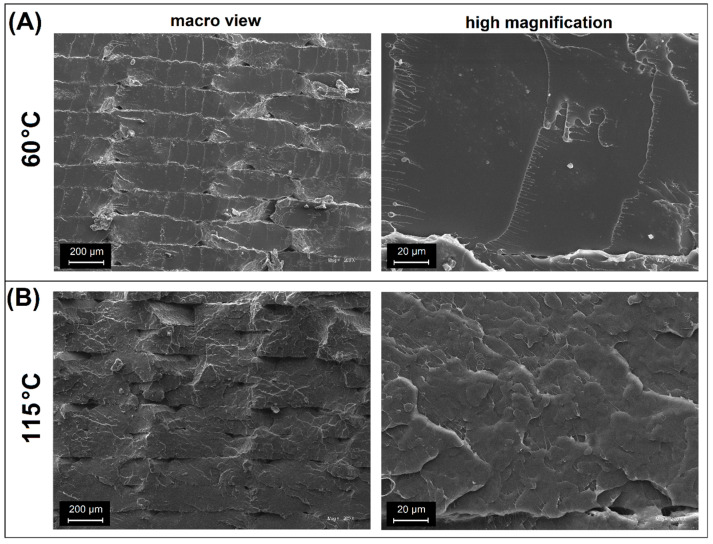
The fracture surface analysis for pure PLA samples: (**A**) materials prepared at 60 °C; (**B**) materials prepared at 115 °C.

**Figure 7:**
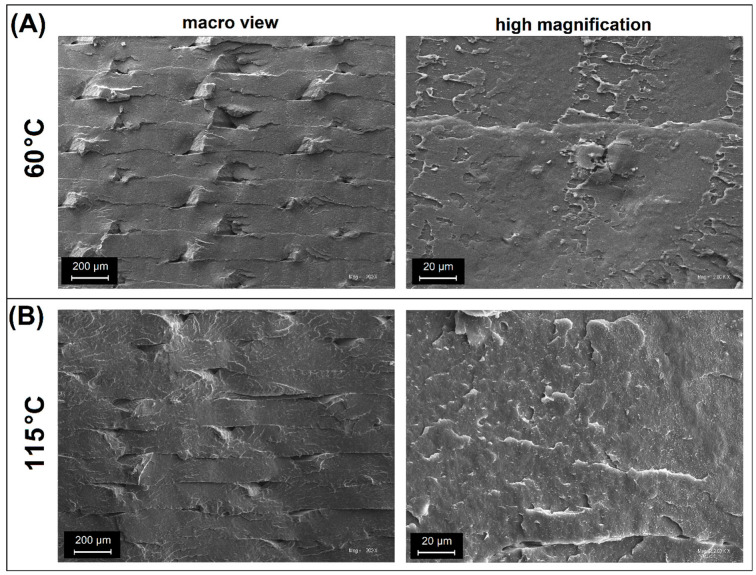
The fracture surface analysis for pure PLA/PHA samples: (**A**) materials prepared at 60 °C; (**B**) materials prepared at 115 °C.

The appearance of the sample microstructure is presented in Figure 6 for unmodified PLA, while Figure 7 reveals the structure of PLA/PHA materials. In both cases, the SEM analysis was conducted for the reference sample printed at 60 °C and for a high bed temperature process at 115 °C. For all scanned samples, observations were conducted at both small (×200) and large magnification (×2000), which helps in evaluating the general structure appearance and micro-sized structure features.

The sample macro appearance for unmodified PLA revealed a typical layered cross-section. The fractured surface is flat with some visible triangular gaps. The presented fracture is typical for amorphous PLA or other types of unmodified polyesters like PETG (polyethylene terephthalate-glycol), PCTG (polycyclohexylenedimethylene terephthalate- glycol), or PET (polyethylene terephthalate) [60,61,62,63,64]. When analyzing the surface at high magnification, it is also clear that on the microscale, the surface of the cracking path is very smooth, which confirms the unfavorable deformation mechanism. The direction of the main crack is a continuous line, without any kinks or plastic deformation, which could increase the amount of energy absorbed by the material during fracture, and thus reduce the brittleness of the material. Due to the characteristics of the 3D printing process itself, it is worth noting the lack of clear traces of delamination in the material layers that comprise the product, which confirms the excellent strength of the created diffusion connections. Not all materials are characterized by this feature. Especially for the FDM process, it is challenging to eliminate delamination for high-temperature polymers or those filled with composite additives [65,66,67,68,69,70,71].

Interestingly, the surface of the PLA(115) sample exhibits a visibly different appearance, where the macro view reveals a more wavy cross-section shape. Compared to the previously observed smooth breakthrough, the presence of a more extensive crack propagation profile indicates different fracture mechanics. The layer boundaries are still clearly marked; however, in selected areas, a lack of separation of the bonded layers can be noticed, which in turn indicates a very good fusion of the deposited layers. The high-magnification appearance revealed another difference compared to the PLA(60) sample, as the roughness at the microscale is visibly higher. The observed structure appearance is very similar to the SEM scans made for highly crystalline polymers like polypropylene or polyoxymethylene [72,73,74,75,76,77,78], which indirectly confirms the ability to improve the PLA crystallinity during the FDM procedure, without postprocessing procedures [32,79,80,81,82,83,84,85,86].

For the SEM scans prepared for PLA/PHA materials, a visible difference is also observed between the structures manufactured at 60 °C and 115 °C. The macro view of the PLA/PHA (60) sample revealed clear boundaries between the deposited layers; however, compared to the unmodified PLA (60) sample, the fractured cross-section is not flat, which suggests that crack propagation was not rectilinear. That behavior may indicate that the influence of the processing method or microstructure inhomogeneity causes some material anisotropies. However, it does not necessarily have a significant impact on reducing the brittleness of the material. The SEM scans prepared at high magnification confirm that, at the microscale level, the inspected surface is very smooth and, in general, similar to that of the unmodified PLA(60) sample. Interestingly, for the PLA/PHA blend, it was not possible to confirm whether the isolated two-phase structure was present. The PLA was confirmed as the main component of the blend [42,43]; however, the morphology and dispersion of the PHA phase were more challenging to distinguish. SEM images confirm the presence of slight structural inclusions. Still, their size and relatively small share in the volume do not allow for an unambiguous determination of the type of component it is. However, the observations confirm that the used PLA/PHA blend is characterized by excellent dispersion and probably partial miscibility, which indicates good compatibilization of the polymer system phases.

After increasing the bed platform temperature for the PLA/PHA(115) blend, the appearance of the macro view is visibly changed. The similarities with the PLA(115) sample are pretty obvious, as the samples produced at high temperatures can easily be observed to have partially disappeared, sharp boundaries between the deposited layer, confirming that higher process temperatures lead to the formation of stronger diffusion connections. The microstructure of the PLA/PHA(115) sample, observed at high magnification, revealed additional roughness that was not observed for the PLA/PHA(60) specimen. The visible changes are again the result of the increase in the crystal structure content; however, the recorded changes are less significant than in the case of the comparison of PLA(60)/PLA(115) samples. In this case, it is challenging to determine whether the formation of the spheroidal structure is specific to the PLA phase or also affects the PHA. In the case of the second polymer, the formation of the crystalline phase is significantly faster, as confirmed by numerous DSC studies [87,88]. Therefore, even at low process or platform temperatures, the PHA crystalline phase is formed without major obstacles; hence, the structural changes observed for the discussed structure are primarily related to the changes in PLA morphology.

Summarizing, microscopic observations clearly indicate that the bed platform temperature, and therefore the thermal conditions for crystallization of the material structure, have a strong influence on the resulting microstructure. For most of the inspected samples, the bonding strength between the part layers was higher, confirming that high bed temperatures significantly improve the interlayer diffusion interactions. This conclusion is quite obvious, but in the case of the discussed PLA-based materials, it leads to clear changes even on a macro scale. More interesting changes are revealed at the microscale, where the improved crystallinity led to a visibly different mechanism of sample crack propagation. In practice, this change affected the results for impact strength measurements, where the increase in impact resistance was visible, especially for unmodified PLA. Structural changes for PLA/PHA blends were less significant, hence the impact strength of the samples is independent of the printing process temperature.

### 3.5. Thermal Properties/Phase Transitions/Crystallinity—Differential Scanning Calorimetry Analysis (DSC), X-Ray Diffraction Measurements (XRD)

**Figure 8:**
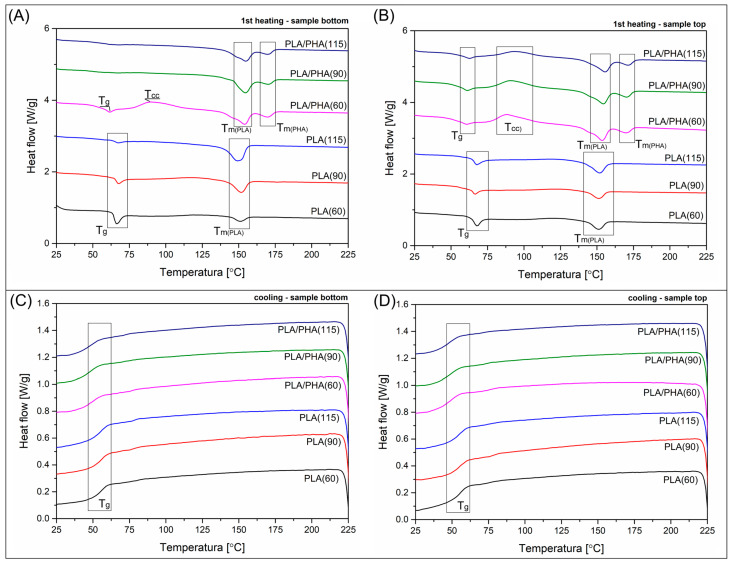
The DSC plots collected from the (**A**,**B**) 1st heating and (**C**,**D**) cooling stages of the measurement. The samples were collected from the top and bottom sides of the specimen.

**Figure 9:**
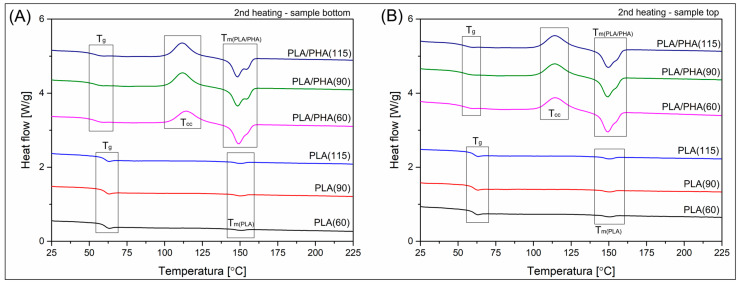
The DSC plots collected from the 2nd heating scan of the measurement. (**A**) the bottom side of the specimen, (**B**) the top side of the specimen.

**Figure 10:**
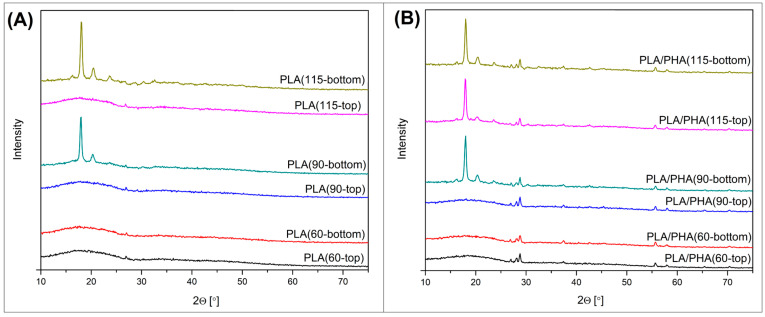
The XDR spectra collected for the prepared specimens: (**A**) PLA; (**B**) PLA/PHA.

Since the main structural differences along the used procedure are correlated with the formation of the crystalline phase, the research study includes differential scanning calorimetry (DSC) and X-ray diffraction (XRD) analysis. The results of the DSC temperature scans are presented in Figure 8 and Figure 9, while the XRD diffractograms are shown in Figure 10. For a more precise evaluation of the DSC analysis results, the most important data collected from the plots are summarized in Table 1. To determine the differences between the top and bottom sides of the sample, both types of measurements were conducted. Each sample was tested twice. For XRD measurements, the test was performed on both the upper and lower surfaces. For DSC tests, the sample placed in the crucible was cut from the outer part (bottom or top) of the 3D printed specimen (dumbbell sample).

The materials used in the study cannot be directly compared; the conducted analysis primarily focuses on evaluating the influence of the processing conditions. In particular, the bed platform temperature. The results presented in Figure 7A reveal the thermal behavior of the bottom part of the samples. Due to the direct contact with the heating plate, the influence of the thermal conditions should be the highest among the other areas of the specimen. The DSC signals for the pure PLA samples were strongly influenced by the increasing bed temperature, as the area under the melting peak of the PLA phase visibly increased. The calculated crystallinity level was growing from the reference value of 4.6% for PLA(60), to 31% for the PLA(specimen), which confirmed that during the bottom layers, the crystallization kinetics were strongly influenced by the growing bed temperature. The value obtained for the PLA(115) sample is very high because no signal deviation was recorded in the DSC graph within the cold crystallization range, which confirms that, for the tested material, further lowering of the table temperature would not have a significant impact on the material’s structural changes. The additional factor that confirms the presence of a more stable macromolecular structure is the less pronounced signal change in the glass transition region, which occurs near 65 °C.

The analysis for the PLA/PHA blend is more difficult since the exact content of the PHA polymer is unknown. For this reason, crystallinity level calculations cannot be conducted; however, some estimation can be made, as the changing thermal conditions strongly influence the melting enthalpy values, which can be directly translated into crystallinity. Additionally, the highly crystalline structure of the PHA phase [87] revealed only a single endothermic peak at around 170 °C, which enables separate analysis for the PLA and PHA phases. In the case of other types of systems, when phase change regions overlap, such comparative analysis is not possible [4]. For the reference sample PLA/PHA(60), the first heating thermogram revealed the presence of typical double-phase structures, as indicated by the presence of two separate exothermic peaks. The position of both peaks remains constant for all tested samples. The temperature of the PLA correlated peak is approximately 155 °C, while that of the PHA component is 170 °C. Interestingly, the cold-crystallization phenomenon signal appears only for the PLA/PHA(60) material, which confirms that for this sample, the formation of the PLA crystalline phase was the least effective compared to the materials printed at 90 °C and 115 °C. It is worth noting that the melting enthalpy value for the PHA phase is almost constant, with recorded values in the range of 9–11 J/g. Without knowledge of the content of this phase, it is difficult to calculate the actual crystallinity. Still, it allows for a clear statement of the lack of significant differences in the kinetics of PHA laminar structure formation under different FDM process conditions. However, the above information allows for a rough calculation of the changes in the melting enthalpy of the PLA crystalline phase, which indirectly provides information on the structural changes within this component of the blend. Taking into account the values of the enthalpy of melting (32.5 J/g) and cold crystallization (26.6 J/g), their difference for the PLA/PHA(60) sample is 5.9 J/g. It can be assumed that this is the base value for the reference sample with a low degree of crystallinity. For both the PLA/PHA(90) and PLA/PHA(115) samples, the melting enthalpy was around 32 J/g, which theoretically gives a more than five-fold increase in the PLA crystalline phase content. Such a change fully explains the substantial changes in the stiffness of the samples in the DMTA tests and the high results of the Vicat tests. Considering that apart from the increase in the content of the PLA crystal structure, also for the PHA phase, we can speak of a stable high crystallinity at the level of 50–60% [43,87], it is possible that apart from the obvious effects of the increase in the content of the PLA crystalline phase, its morphology is also of great importance. For the discussed materials, where structural studies revealed a very small size of PHA phase inclusions, it is possible that they act as an effective enucleation center for the PLA crystalline phase, which translates into the observed kinetics of crystallinity changes.

The DSC plots presented in Figure 8B reflect the same 1st heating, but for specimens taken from the top layer of the dumbbell sample. It is clear that for unmodified PLA, the appearance of the DSC plots suggests a lack of significant difference between the investigated specimens. The calculated crystallinity was only slightly influenced by the bed platform temperature, since the initial Xc for the PLA(60) sample was around 11%. At the same time, for the PLA (115) material, it reached 17%, which fact strongly confirms that even for relatively thin 4 mm specimens, there are significant differences in thermal conditions during the FDM printing process. A similar correlation is observed for PLA/PHA-based materials, where the appearance of separated endothermic peaks is still present; however, for all of the investigated materials, the presence of cold crystallization phenomena confirms the low content of the PLA crystalline phase. Again, the presence of unknown content of the PHA component makes the correct calculations more complex. The calculated difference between the melting and cold crystallization enthalpy values (ΔHm − ΔHcc) makes it possible to calculate the relative difference in PLA crystallinity. For the low bed temperature processing PLA/PHA(60) the calculated values are close to 6.5 J/g, while when increasing the Bed temperature to 90 °C and 115 °C, the enthalpy values are 8.8 J/g and 14.2 J/g, respectively. These values are significantly lower than the results of 32 J/g for the bottom layer area, confirming the large difference in crystallinity level between different areas of the prepared parts.

The analysis of the DSC cooling scans revealed no differences between the thermograms collected in Figure 8C,D. The thermal history was primarily influenced by the FDM processing conditions, such that after remelting the crystalline structure, the presented thermograms are influenced only by the blend composition. Interestingly, the only remarkable signal that appears during the cooling stage is related to the glass transition of the PLA phase. No clear exothermic signal was observed for any of the samples, which could suggest the formation of a crystalline phase. Only a small peak around 80 °C suggests the occurrence of some phenomenon, but due to the minimal scale of these phenomena, they cannot be considered significant structural changes.

In the case of polyester materials, the dominant character of the amorphous phase allows for a more detailed analysis of the glass transition, which is, however, not possible for both tested materials, since the PHA phase T_g_ is usually postponed to −30 °C, which is below the range of the presented DSC analysis [89]. Nevertheless, the presence of the PHA phase appears to influence the changes in the nature of the glass transition for the main component of the blend, i.e., PLA. The first observation concerns the visible reduction of T_g_ towards lower temperatures, to about 55 °C, compared to the original temperature of 65 °C for PLA. Potentially, this change could be caused by the use of a different PLA variety in the mixture; however, a shift of 10 °C is too significant a change considering the differences in the molecular weight of the base polymer. Therefore, the leading cause should be sought in the shift in the mobility of the amorphous phase of PLA caused by partial miscibility with PHA. In the case of the examined samples, it is also worth mentioning that, in addition to changes in the position of the T_g_ inflection point, significant differences in the appearance of the curve in the transition region are also visible. This fact is partially related to the increase in the crystallinity level of the samples, but also to the change in the macromolecule relaxation energy for samples produced at different temperatures [90]. Here, this phenomenon is evident for unmodified PLA, where, beyond the inflection point, a clear relaxation peak is observed, smaller for high printing temperatures, which is particularly visible for samples taken from the lower part of the dumbbell specimen. In the case of PLA/PHA samples, the changes in the relaxation enthalpy are less smooth, because only for PLA/PHA(60) a clear DSC signal can be noted in this region. In contrast, for samples printed at 90 °C and 115 °C, the T_g_ region has a very indistinct signal change.

Due to the reversible nature of the glass transition phenomenon, the changes observed during 1st heating stage are analogous in the cooling graphs. However, as a result of structure remelting and erasing the thermal history resulting from the different 3D printing process conditions, the differences observed during the 1st heating are no longer visible, and the graphs for all PLA and PLA/PHA samples are identical, regardless of the initial printing temperature. Since the relaxation is categorized as a non-reversible phenomenon, the differences observed at the 1st heating stage cannot be recorded for remelted samples. Hence, similarly to the crystalline phase content, the size of the relaxation peak area for the tested materials in the further stages of the DSC analysis depends only on the type of material used.

The 2nd heating thermograms are presented separately in Figure 9. The results reflect the thermal properties of the remelted macromolecular structure. For this reason, it is possible to examine the thermal properties without the influence of the processing conditions, which allows revealing the main thermal differences between the filament types. As for previous tests, the heating rate was set to 10 °C, a typical value for the DSC heating stage. However, from an analytical point of view, the speed of the cooling stage preceding the heating stage was more critical, with a rate of 10 °C/min. This information is important because for thermoplastic polyester-based materials, the cooling rate achieved during the standard DSC test (5–20 °C/min) is usually too high to form the crystalline phase, which is particularly true for slowly crystallizing PLA varieties. The second heating scans for unmodified PLA reveal almost identical results for all tested specimens, with no visible difference due to the original thermal history of the material. The absence of a clear cold crystallization signal and a minimal melting enthalpy peak area confirmed the highly amorphous character of the used PLA type. As a result of remelting the material structure, the DSC signals for the sample taken from the top and bottom of the dumbbell specimen are identical. In the case of the observed curves, the dominant signal of phase transitions is the inflection of the graph in the glass transition temperature range (T_g_), and the signal of melting of the crystalline phase is minimal. Considering that the enthalpy value ranges from 1.2 J/g to 2.8 J/g, the content of the crystalline phase in none of the samples exceeded 3%.

The 2nd heating scan for the PLA/PHA material revealed the exact amorphous nature of the remelted samples; however, in contrast to unmodified PLA, the formation of a large area cold crystallization peak (T_cc_) and consecutive melting peak confirmed the enhanced nucleation potential for this type of blend. Interestingly, the appearance of the melting area of the curve strongly differs for the 1st heating plots. The initial separate peaks for the PLA and PHA phases have merged into a single peak with two maximum points. The first maximum, at 148 °C, corresponds to the melting of the PLA phase, and the second maximum, recorded at 155 °C, is associated with the PHA phase. Such a significant change in the position of the PHA melting peak cannot be attributed to the standard error of the method. It is possible that, in the discussed case, the influence of the properties is due to the high compatibility of the mixture components or their partial miscibility. In the case of the FDM process, the period of formation of the crystal phase domains for both types of materials was relatively long, because the minimum temperature of the work table is 60 °C, and each subsequent polymer layer heats the previous one to a temperature significantly above the cold crystallization temperature range. Such conditions favor the formation of stable crystal formations, which increases their melting temperature, as in the discussed case [91].

Considering the data collected during DSC measurements, it is worth noting that the stability of the crystal structure of the obtained materials is secondary in nature and likely results from the formation of lamellar structures during slow cooling induced by the high temperature of the working table. An additional thermal factor is undoubtedly the phenomenon of cyclic reheating of the polymer structure by new layers of material applied to it, which phenomenon is often described in the literature, both in simulation models and experimental studies [92,93,94].

The results of the XRD studies presented in Figure 10A reveal the spectra for unmodified PLA specimens, similar to the DSC; the chart collects the results for the top and bottom sides of the 3D printed part. It is clear that the results strongly support the conclusions from the previously described DSC analysis, since the crystallinity differences are mostly associated with the bed platform temperature and the measurement position. For all of the PLA samples tested at the top position of the specimen, the XRD spectra revealed only a single broad peak within the range of 10° and 30°, which corresponds to the typical result for the amorphous type of polymers [81,95,96]. Taking into account that in the case of DSC measurements, the results indicate a level of crystallinity of about 12%, it is worth noting that this is the result for measurements of a sample about 1 mm thick, collected from the top layer of the print, where the influence of the heat of the build platform and subsequent applied layers is significant. XRD measurements are carried out on the surface; therefore, the impact of thermal processes on the last layer of the model lasts only a few dozen seconds. Hence, even for the PLA(115) sample, the structure of the top layer of the product shows an amorphous character. A significantly different appearance of the spectral plots is observed for the measurements of the lower surface of the sample. This time, the amorphous structure of the model is visible only for the PLA(60) sample, while the materials printed at 90 and 115 °C exhibit significant structural changes. Two sharp peaks recorded at 2ϴ of 17.1° and 19.3° correspond with (110)/(220) and (203) planes, indicating the dominating α-phase structure. Interestingly, for the PLA(115-bottom) specimen, it is possible to distinguish small additional peaks from other crystallographic planes, which confirms the highest crystallinity level for samples printed at 115 °C.

When analyzing the PLA/PHA material, it is evident that all samples display new peaks in the XRD spectra within the 2θ range of 25–30°. The presence of these peaks is associated with the formation of the PHA crystalline phase, which can be attributed to the (101) and (111) planes [42,43]. This fact confirms that the formation of the PHA crystalline phase is not determined by the FDM process parameter, or at least that the standard parameter of 60/210 °C bed/nozzle temperature is sufficient for the growth of the PHA lamellar structure. Similarly to the other PLA-based materials for the low-crystalline specimen, the amorphous halo region was observed in a broad range of 10–30°. It is clear that for the PLA/PHA(60) sample, both investigated surfaces are characterized by a low crystallinity level of the PLA phase. Similar results were also obtained for the PLA/PHA(90-top) sample, where the thermal conditions for the upper surface of the dumbbell specimen were insufficient to obtain significant structural changes. Visible changes, similar to samples based on unmodified PLA, are observed only for the material from the bottom part of the sample PLA(90). However, these changes are analogous to the results obtained for pure PLA. The behavior of the PLA/PHA sample (115) is noteworthy, as both measurement variants (top and bottom) yield a clear peak signal for the (110)/(220) and (203) planes, indicating the presence of highly crystalline PLA structures. The results confirm the improvement of the crystallization kinetics of the PLA phase in the case of PLA/PHA blends.

## 4. Discussion

The presented results are a summary of the preliminary research study conducted to investigate the potential use of PLA/PHA blends at elevated temperature conditions. The research methodology was designed to determine if modifying the parameters of the FDM printing process, within the capabilities of standard desktop devices, enables altering the thermal conditions and resulting in beneficial structural changes in the processed material. One of the first conclusions resulting from mechanical measurements for the tested materials allows us to state that, unlike unmodified PLA, the changes observed in the PLA/PHA system are slightly more complex than the typical PLA correlations related to the increase in the crystalline phase content, for samples made of unmodified PLA, the change in platform temperature from 60 °C to 115 °C results in a direct improvement in both tensile and flexural modulus, as well as strength. The changes amount to approximately 10–15%, which confirms the standard correlation between the increase in PLA crystalline structure content and the results of mechanical measurements for materials subjected to this type of modification. For PLA/PHA materials, the changes are not so obvious, as the increase in stiffness and strength was not recorded in any of the tests. This behavior indicates that the PLA/PHA mixture has a two-phase or partially miscible system, as a result of which the morphology of the PLA phase does not determine the mechanical properties. Unfortunately, this aspect of the research has not been clearly explained, despite a fairly wide range of complementary studies. Both the homogeneity of the structure, the small size of PHA phase inclusions, and the fact that the presence of PHA induces the growth of the PLA crystalline phase contribute to the potential for increasing stiffness in samples produced at elevated thermal process conditions. However, as it turns out, certain structural dependencies in the produced materials cause negative changes in stiffness despite an increase in the crystalline phase content.

This phenomenon seems interesting and worth explaining; therefore, as part of the planned supplementary studies, a series of tests will be prepared for samples obtained under annealing conditions and solid products obtained by injection molding, this procedure will aim to compare the properties of solid materials and those with a homogeneous structure, which will eliminate the negative factors related to the low sorption of the additively manufactured structure.

## 5. Conclusions

In terms of key thermomechanical properties, it is evident that PLA/PHA materials have an advantage over standard PLA. In particular, the thermomechanical Vicat (VST) test indicates that even for materials printed at 90 °C, the increase in the recorded softening temperature was approximately 80 °C, from 58 °C to 138 °C. Notably, the DMTA showed that the change in viscoelastic properties for reference materials in the case of PLA/PHA samples occurs at much lower temperatures, which influences the changes in properties observed after the printing process; the DSC measurement results also confirmed this. The possibility of achieving a sufficiently high degree of crystallinity in the material structure was feasible at temperatures well below 100 °C, which served as the starting point for the changes observed in the unmodified PLA samples.

The above studies have allowed us to indicate several important directions for further work, where research is planned to prepare composite materials with the addition of mineral fillers. In the discussed cases, talc has the most significant potential for application due to its nucleation ability. In the case of further work, much greater emphasis will be placed on performing a precise analysis of the deformation of complex-shaped samples, and printing tests will also be carried out under controlled temperature conditions of the printing chamber.

## Figures and Tables

**Table 1:** The basic thermal properties collected from the DSC plots.

Sample	Peak Position [°C]		Enthalpy [J/g]		Crystallinity (for PLA) * [%]
	Cold Crystallization	Melting	Cold Crystallization ΔH_cc_	Melting ΔH_m_	
Top side of the specimen					
PLA(60)	119	151	3.3	14.9	12.3
PLA(90)	120	151.5	2.2	12.6	11
PLA(115)	119.5	152	3.8	19.8	16.9
PLA/PHA(60)	88	153.5/170	27.6	34.1	-
PLA/PHA(90)	91	154.5/170.5	25.1	33.9	-
PLA/PHA(115)	94	155.5/170.5	17.8	32.0	-
Bottom side of the specimen					
PLA(60)	125.5	151	2.8	7.5	4.9
PLA(90)	12.5	152	6.6	19.4	13.5
PLA(115)		149	-	31.1	32.8
PLA/PHA(60)	88.5	154/170	26.6	32.5	-
PLA/PHA(90)	-	154.5/170	-	32.0	-
PLA/PHA(115)	-	155/170	-	31.5	-

* The crystallinity calculations are limited to pure PLA samples.

## Data Availability

The original contributions presented in this study are included in the article. Further inquiries can be directed to the corresponding author.

## Abbreviations

The following abbreviations are used in this manuscript:

FDM	Fused deposition modeling
PLA	Poly(lactic acid)
PHA	Polyhydroxyalkanoate
HDT	Heat deflection temperature
VST	Vicat softening temperature
DMTA	Dynamic mechanical thermal analysis
DSC	Differential scanning calorimetry analysis
TGA	Thermogravimetric analysis
SEM	Scanning electron microscopy method
XRD	X-Ray diffraction analysis
